# Genetic Differentiation of Chinese Fir Populations From Mainland China and Taiwan as Revealed by Genotyping‐By‐Sequencing Analysis, With Implication for Taxonomic Position of the Species

**DOI:** 10.1002/ece3.71270

**Published:** 2025-04-11

**Authors:** Yajing Zhang, Yangyang Sun, Minchen Zhong, Fenglin Chen, Yaning Wang, Mulualem Tigabu, XiangQing Ma, Ming Li

**Affiliations:** ^1^ College of Forestry Fujian Agriculture and Forestry University Fuzhou China; ^2^ Chinese Fir Germplasm Innovation Engineering Research Center of Fujian Province Fuzhou China

**Keywords:** Chinese fir, Genetic Diversity, Genotyping by Sequencing (GBS), Population Structure, Single‐Nucleotide Polymorphism (SNP) Discovery

## Abstract

Climate change and strait isolation during the glacial period had a profound effect on the differentiation of gymnosperms on both sides of the Taiwan Strait. The taxonomic status and population structure of *Cunninghamia konishii* (Taiwan) and *C. lanceolata* (mainland China) remain contentious due to conflicting morphological and molecular evidence. Thus, we sampled 92 accessions from seven natural populations, six from mainland China and one from Taiwan, and conducted high‐throughput genotyping‐by‐sequencing (GBS) analysis. The northern marginal population exhibited the lowest genetic diversity (*θπ* = 4.828 × 10^−3^), while the Taiwan population had the highest (*θπ* = 5.821 × 10^−3^), reflecting its role as a glacial refugium, while mainland populations retained lower diversity due to post‐glacial bottlenecks. There was little difference in Tajima's D values of selection pressure between mainland China and Taiwan. However, significant gene flow (*Nm* = 2.839) was observed, combined with low *F*_*ST*_ values (0.072–0.122), which indicate low genetic differentiation among *C. lanceolata* and *C. konishii*. Migration analysis indicated a high probability of unidirectional gene flow from mainland China to Taiwan, with the Dongshan Land Bridge facilitating pre‐glacial gene flow. We conclude that *C. konishii* represents an ecotype of *C. lanceolata* , shaped by environmental plasticity and incomplete isolation. This study enhances our understanding of the gene flow and evolutionary processes shaping the species and offers new insights into their taxonomic classification.

## Graphical abstract

This study investigates the genetic diversity and taxonomic status of *Cunninghamia lanceolata* and Cunninghamia konishii, exploring their differentiation due to climate change and glacial isolation. Genotyping‐by‐sequencing (GBS) analysis of 92 accessions revealed minimal genetic differentiation and significant gene flow from mainland China to Taiwan, suggesting that *C. konishii* is an ecotype of *C. lanceolata* , rather than a distinct species.

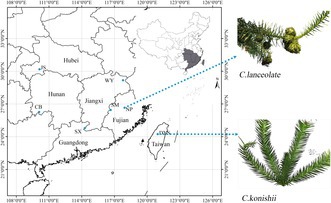

## 1 Introduction

The connection and isolation between Taiwan Island and mainland China during the Quaternary glacial and interglacial periods had a significant effect on the geographic distribution pattern, population structure, and species differentiation of plant groups on both sides of the Taiwan Strait. Many geological and archaeological data have proven the existence of a land bridge called the “Dongshan Land Bridge” in the Taiwan Strait extending from Dongshan Island in Fujian Province, passed through the Taiwan Strait shoal to the Penghu Islands, and ultimately connected to the west shoal of Taiwan (Lin 1982; Zhao et al. 2017; Zhao et al. 2018). During the great Ice Age, the expansion of glaciers caused the sea level to fall, and the Dongshan Land Bridge emerged to connect Taiwan Island with mainland China (Kitamura 2016). The East Asian continental plants, especially gymnosperms, migrated from north to south to low‐altitude mountainous areas and took refuge on Taiwan Island through the Dongshan Land bridge (Zhao et al. 2017). Sea level rise causes temperatures to rise during interglacial periods, which causes remigration from southern refuges to the north and, ultimately, geographic isolation of populations on the mainland and islands (Zhao et al. 2018). Plant responses to environmental variability and climate change across the Taiwan Strait are characterized by migration (or changes in geographic distribution) and adaptive evolution (or formation of new species). The climatic influence during glacial and interglacial periods, as well as the isolation of the Taiwan Strait, might have caused the disruption of intraspecific gene flow. This led to evolutionary differences between Chinese fir populations in mainland China and Taiwan.

**FIGURE 1:**
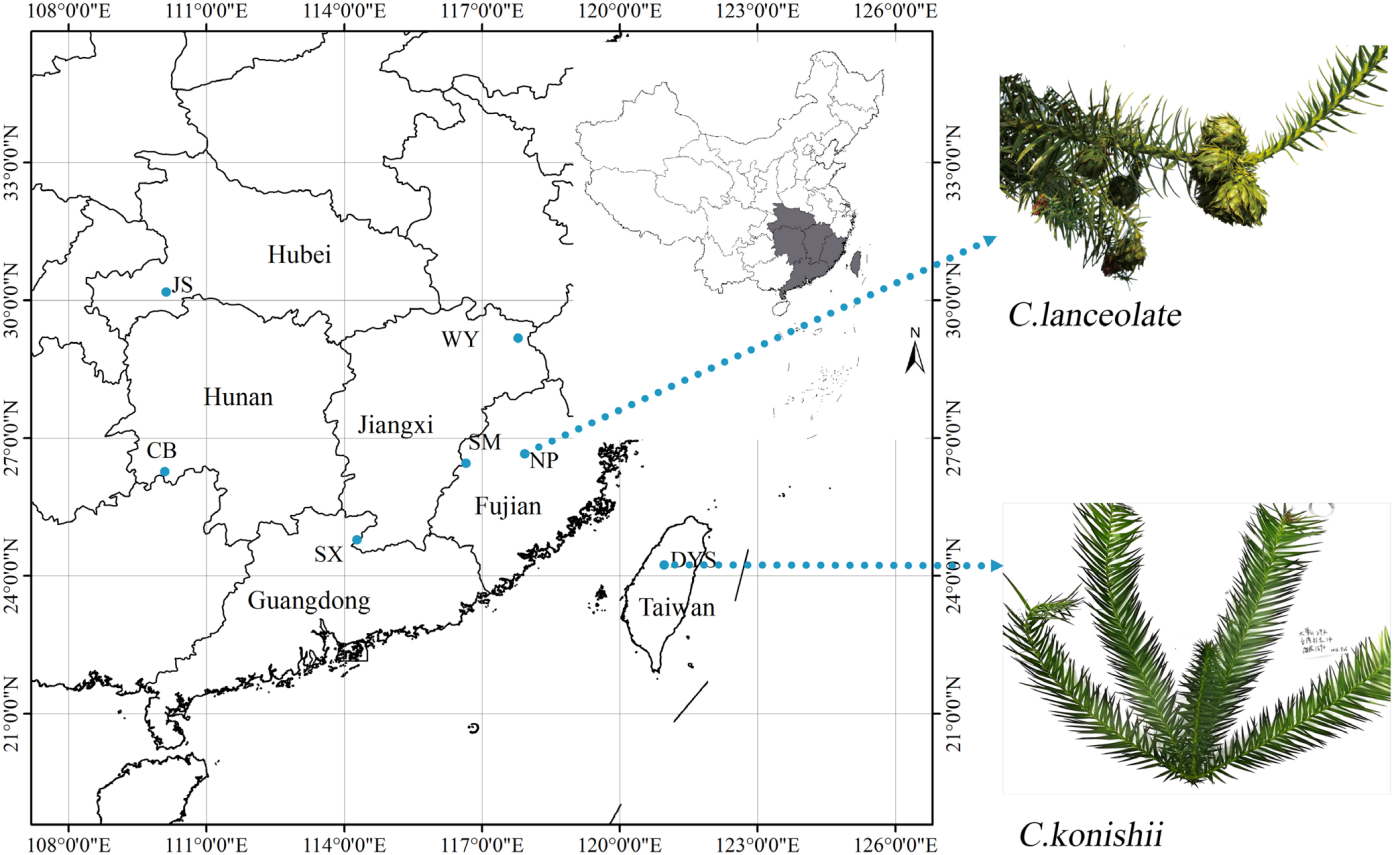
Geographic distribution and leaf phenotype of the sampled populations.

*Cunninghamia lanceolata* (Lamb.) Hook belongs to the Cupressaceae family, commonly known as Chinese fir, and is a species distributed on both sides of the Taiwan Strait. It exhibits typical migratory and adaptive evolutionary responses to this historical process. The fossil records of *Cunninghamia* indicate that the genus originated in the northeastern region of East Asia during the Early Cretaceous and was then widely distributed in temperate and subtropical regions of the Northern Hemisphere, including Europe and North America, from the Eocene to the Pleistocene (Shi et al. 2014). Due to the influence of the Quaternary Ice Age, *C. lanceolata* has become extinct in most areas and is now primarily distributed in southern mainland China, Taiwan, and northern Vietnam. It has become the most commercially valuable coniferous species in China (Yan et al. 2021). Chinese fir occurring in both mainland China and Taiwan has distinct morphological differences (Figure 1). Chinese fir distributed in Taiwan has shorter and narrower leaves, stomatal bands on the sides, smaller cones, and earlier flowering (Chung et al. 2009). Based on these morphological differences, some taxonomists believed that it was either an independent species or gradually evolving into a new species and named it *Cunninghamia konishii* Hayata (Hwang et al. 2003; Lin 2007). However, many scholars generally believe that this taxon is a variety of the Chinese fir, named *C. lanceolata* var. konishii (Hayata) Fujian. Studies using ISSR and SSR molecular markers also revealed a small genetic differentiation between these species (Chen et al. 2017; Chung et al. 2011).

Similarly, studies on the relationship and geographic distribution of Chinese fir in mainland China and Taiwan revealed that *C. konishii* evolved from *C. lanceolata* and has gradually become a new species (Chung et al. 2004). Furthermore, the cpDNA markers were used to study the phylogeography of 64 wild Chinese fir individuals in Taiwan. The study concluded that the Chinese fir population migrated from mainland China during the Ice Age through long‐distance seed dispersal (Hwang et al. 2003). Using leaf fatty acids and volatile components, as well as the chlL gene and 18SrRNA gene markers, the phylogenetic relationship between *C. konishii* and *C. lanceolata* was analyzed, which revealed a very close relationship (Lin 2007). Using ISSR markers, Chen et al. (2017) found that the genetic diversity of natural populations of *C. lanceolata* from Fujian province in mainland China was higher than that from Taiwan. However, the ISSR, SSR, and cpDNA methods have a limited number of marker sites and lack sufficient resolution to accurately reflect the level of species differentiation in Chinese fir (Zheng et al. 2019).

With the continuous development of next‐generation sequencing (NGS) technology, it is now possible to provide a high‐throughput and cost‐effective molecular tool for various molecular biology research (Wu and Blair 2017). However, the application of high‐throughput sequencing technology for many nonmodel species is still limited due to the lack of reference genomes. This is particularly true for a large number of forest tree species with a big genome size. Genotyping by sequencing (GBS) reduces the complexity of the genome through high‐throughput sequencing of tags generated by enzymatic digestion. It is a simplified method for rapidly sequencing the entire genome to identify high‐density single‐nucleotide polymorphisms (SNPs) without the need for a reference genome. This method is widely used for various purposes such as developing molecular markers, studying species diversity, analyzing population genetics, constructing genetic maps, and mapping quantitative trait loci (QTL) (He et al. 2014; Küpper et al. 2018). Currently, GBS has been successfully applied to study the population genetic diversity and evolution of *Pinus radiata* , *Pinus sylvestris* , *Picea engelmannii* , and other conifers (Gamal El‐Dien et al. 2015; Hall et al. 2020; Holliday et al. 2019; Pan et al. 2015). To the best of our knowledge, no study has directly compared the population genetic structure of Chinese fir in mainland China and Taiwan using GBS to generate SNP markers, primarily because of the lack of whole‐genome data. There have been reports of using the specific locus‐amplified fragment sequencing (SLAF‐seq) method to explore a large number of SNPs in order to establish a high‐density SNP panel for the Chinese fir advance breeding population. Additionally, SNPs have been used for association studies with growth‐related traits and secondary metabolite contents in red‐ and white‐heart Chinese fir (Cao et al. 2022; Su et al. 2016; Zheng et al. 2019). However, most of the research on the genetic diversity of Chinese fir is based on low‐resolution molecular markers, with limited consideration of population geographic origins and sample size. In particular, there is a lack of germplasm resources from wild populations (Chen et al. 2017; Li et al. 2017; Lin et al. 2020).

Thus, we utilized the GBS method to acquire extensive SNP data in order to uncover the genetic diversity and structure of natural populations of Chinese fir in mainland China and Taiwan. Genetic diversity is a prerequisite for maintaining populations as evolutionarily viable units that can adapt to changing environmental conditions in the long term (Yang et al. 2019). Evaluation of the level and structure of genetic variation and differentiation in natural populations facilitates the effective use of these valuable genetic resources in future breeding to broaden the genetic diversity and genetic resource conservation programs (Alipour et al. 2017). Because of the long period of adaptive differentiation, cross‐pollination characteristics, geographic isolation, and other factors, the morphological characteristics and adaptive capacity of Chinese fir exhibit high variability, leading to genetic variation across different distribution areas (Lin et al. 2020). Unfortunately, due to the extensive period of artificial introduction and cultivation of various provenances, as well as the depletion of natural populations, there is currently a scarcity of natural Chinese fir forest germplasm resources available for collection (Chen et al. 2017; Duan et al. 2017; Li et al. 2017; Lin et al. 2020). Our aim was to analyze and compare the genetic differentiation and diversity on both sides of the Taiwan Strait. For this purpose, we sampled 92 accessions from seven natural populations, including six populations from mainland China and one population from Taiwan. The specific objectives of this research were to utilize GBS technology (1) to assess the genetic variability of native Chinese fir populations, (2) to examine the genetic differentiation and structure of Chinese fir populations in mainland China and Taiwan, and (3) to determine whether *C. konishii* is a distinct species or a subspecies of *C. lanceolata*.

## 2 Materials and Methods

### 2.1 Plant Material and DNA Isolation

We collected 92 samples from seven natural populations, including six from mainland China and one from Taiwan. These samples were selected from wild populations that have been confirmed as ancient Chinese fir groups, representing local germplasm resources (Figure 1). The mainland China populations are representative of the core distribution area that stretches from Shixing, Guangdong province (24.75°N, 111.50°E) to Jianshi, Hubei province (30.18°N, 110.12°E). In these natural populations, the mean annual temperature varies from 10.2°C to 19.6°C, and the elevation spans from 153 to 1207 m a.s.l. The average elevation of the Taiwan population is 2250 m, and the annual precipitation is 4000 mm, which is significantly higher than that of mainland China populations. A minimum distance of 100 m between sampled trees was maintained for random selection and collected samples from different geographic locations within each population to maximize spatial separation and reduce the impact of genetic correlation on the research results. The individual and morphological hand‐drawn pictures of sampled *C. lanceolata* and *C. konishii* are shown in Figures S1 and S2.

In terms of geographical representation, 15 individuals were collected from Hunan province (CB), Hubei province (JS), Nanping (NP), and Sanming (SM) each, and 13 from Jiangxi province (WY) and 10 from Guangdong province (SX) due to variability in availability of individuals. These six populations were considered *C. lanceolata* . Because the natural population of *C. konishii* in Taiwan is rare and it is close to the distribution area of Chinese fir introduced to Taiwan from mainland China. To ensure the accuracy of sampling and the distance between individuals, we only collected one confirmed population of 9 individuals from Dasyueshan National Forest Recreation, which was considered to be *C. konishii* Hayata. More details on the geoclimatic conditions of each group are listed in Table 1.

**TABLE 1:** Geographic locations and climatic conditions of the Chinese fir populations.

Population	Geographical location	Latitude (N)	Longitude (E)	Sample size	Elevation (m)	Annual temperature (°C)	Annual precipitation (mm)
GD	Shixing, Guangdong Prov.	24°46′56”	114°16′24”	10	663	19.6	1825
HN	Chengbu, Hunan Prov.	26°15′48”	110°05′24”	15	834	16.1	1218
HB	Jianshi, Hubei Prov.	30°10′48”	110°07′03”	15	1207	10.2	1550
JX	Wuyuan, Jiangxi Prov.	29°10′24”	117°46′59”	13	168	17.8	1488
NP	Nanping, Fujian Prov.	26°39′16”	117°55′41”	15	153	19.4	2000
SM	Sanming, Fujian Prov.	26°27′00”	116°38′58”	15	590	16.0	1800
TW	Dasyueshan, Taiwan	24°13′47”	120°57′53”	9	2250	12.4	3800

Leaf samples were collected and brought back to the lab, dried with silica, and stored at 4°C for DNA extraction. Total genomic DNA was extracted from the samples using a UNIQ‐10 Plant Genomic DNA Preps Kit (Sangon, Shanghai, China). The DNA was quantified using an epochTM microplate spectrophotometer (BioTek, Winooski, VT, USA) and diluted to a working concentration of 30 ng/μl.

### 2.2 Genotyping by Sequencing and SNP Filtering

GBS library and bi‐allelic SNP calling were conducted by Guangzhou Gene Denovo Bio‐Technology in Guangzhou, China, according to an established method (Elshire et al. 2011). GBS involves five major steps: sample preparation, library assembly, sequencing, SNP calling, and diversity analysis (Taranto et al. 2016). Briefly, the genomic DNA of 92 accessions was digested with the restriction enzyme EcoRI‐NIaIII and then ligated with a barcode adaptor and a common Illumina sequencing adaptor. The DNA fragment of the adaptor was purified using Agencourt AMPure XP beads (Beckman Coulter), and a fragment within the range of 300–400 bp was selected for PCR amplification (Rohland and Reich 2012). The constructed library was purified, tested, and sequenced on the HiSeq X10 PE150 (Illumina Inc., San Diego, CA, United States) using standard Illumina protocols and kits.

The raw sequence data were processed through the GBS discovery pipeline in TASSEL software (version 3.0) using the default settings (Glaubitz et al. 2014; Lu et al. 2013). The FASTQ raw files and sample key files, containing information about the plate layout and barcodes for each sample, were utilized to create a GBS database for SNP calling (Wu and Blair 2017). Only the sequences that had perfect barcode matches, followed by the sticky end sequence of an EcoRI‐NIaIII restriction enzyme cut site, were identified and retained. Reads that had no matching barcode or cut site remnant were excluded from the analysis. Additionally, reads containing unidentified bases (N), adapter dimers, and low‐quality reads (Q≤ 10 accounting for more than 50%) were also excluded. After removing barcodes and common adapters, the sequences were trimmed to a length of 142 bp. The clustering software Stacks (version 1.43) was used for clustering of high‐quality clean reads (Rochette and Catchen 2017); the clustered tags are overlapped, and the tags without overlap are linked by N, and then scripted into a pseudo‐genome of consistent size for subsequent analysis. The unique sequence tags with a perfect match to the pseudo‐genome were called SNP discovery. The raw sequencing data yielded an average depth of 20 × per sample at SNP loci after filtering; this depth is consistent with GBS studies in other large‐genome conifers (e.g., *Picea mariana* , Pavy et al. 2016), where 10–20× depth is standard for reliable SNP calling. Low‐depth loci (< 10×) were excluded during filtering, and loci with > 20% missing data across samples were removed. SNPs with heterozygotes < 10%, a minor allele frequency > 1%, and missing data < 20% were used for further analysis. All newly discovered SNPs were scored for coverage, depth, and genotypic information for genetic diversity and population structure analysis.

### 2.3 Genetic Diversity and Population Structure Analysis

The average pairwise divergence within a population (*θπ*) and the Watterson's estimator (*θw*) were calculated using the PopGen package (https://cran.r‐project.org/src/contrib/Archive/popgen/) and the BioPerl module (Mace et al. 2013). Tajima's D analysis was used for neutral tests by comparing the two mutation rates of *θπ* and *θw* to detect the positive selection effect (Chong et al. 2016). *FST* and *Nm* were calculated by determining the individual differences in the average number of SNPs within and among populations using the PopGen package. The genetic distance tree analysis was performed using the neighbor‐joining method with the software TreeBesT 1.9.2, based on the SNPs information. SNPs were used to calculate the genetic distance between individuals. A distance matrix was generated using the Plink toolset for whole genome association analysis and genome‐wide complex trait analysis. Principal coordinate analysis (PCA) was performed on all individuals using this distance matrix.

To further analyze possible evolutionary patterns between Chinese and Taiwanese populations, we grouped the six populations from mainland China into one population, with the TW population as the other population. We used the Migrate software to estimate the direction of gene flow between mainland China and TW (Beerli and Palczewski 2010). We considered three models: a complete model with two population sizes and two migration rates (from mainland to TW and from TW to mainland); a model with two population sizes and one migration rate from mainland to TW; and a model with two population sizes and one migration rate from TW to mainland (Han et al. 2015; Zhiduan et al. 2013). The marginal likelihood of each model was evaluated using the thermodynamic integration method. In each model, a minimum of four heating chains was utilized to predict the optimal results using the recommended temperature scheme by setting the number of steps recorded in the chain to 100,000 and the replication parameter to 3 and retaining the default values for other parameters. Finally, the marginal likelihood of all models was compared in order to infer the direction of gene flow (Han et al. 2015).

The population structure analysis was conducted using the parametric Bayesian model‐based clustering method implemented in the ADMIXTURE program (http://software.genetics.ucla.edu/admixture/). ADMIXTURE is a software tool that allows for maximum likelihood estimation of individual ancestries from multilocus SNP genotype datasets (Alexander and Novembre 2009; Gras and Grosu 2019). To determine the number of populations (K) that represent the main structure in the data, we conducted 100,000 runs with a burn‐in period of 50,000 Markov Chain Monte Carlo iterations. Seven independent runs were performed for each simulated value of K, ranging from 2 to 101. Subsequently, a Pr (X|K) index was used to calculate ΔK for each K value, following the formula described by Evanno et al. (2005). The optimal K value depends on the first peak of Ä ΔK = |L′′(K)|/s[Pr(x|k)], where |L′′(K)| denotes the absolute value of the second‐order rate of change of Pr(X|K), and s[Pr(x|k)] denotes the standard deviation of Pr(X|K) (Li et al. 2017). The Mantel function in the VEGAN package of RStudio 0.98.1091 was used to conduct a Mantel test. The correlation between the genetic distance matrix and the geographic distance matrix of different geographic groups of Chinese fir was analyzed.

## 3 Results

### 3.1 Morphological Analysis

To evaluate the taxonomic distinction between C. konishii (Taiwan) and *C. lanceolata* (mainland China), we conducted a comparative morphological analysis of key traits, including bark, leaves, cones, and bract scales, based on field observations and herbarium specimens (Figures 1, S1 and S2). While subtle phenotypic variations were observed (Table 2), these differences were not statistically significant and likely reflect environmental plasticity or localized adaptation rather than species‐level divergence. The bark color of *C. konishii* ranges from light reddish‐brown to dark brown, with a rough, fissured texture, and the bark of *C. lanceolata* is predominantly grayish‐brown with reddish undertones, displaying similar fissuring patterns. The leaves of *C. konishii* are narrowly linear‐lanceolate (1.5–2.5 mm wide, 1.5–2 cm long), slightly falcate (sickle‐shaped), and radially arranged on shoots. The adaxial (upper) surface is glossy deep green. The leaves of *C. lanceolata* are broader (3–5 mm wide, 2–6 cm long) and lanceolate, typically arranged in two ranks on lateral branches, and the adaxial surface is matte green. The cones of *C. konishii* are broadly ovate (2.5–3.5 cm long), with leathery, ovate bract scales bearing a slightly acute apex and less conspicuous serrulate margins. The cones of *C. lanceolata* are oval (3–4 cm long), with triangular‐ovate bract scales that have a pronounced spiny cusp and irregularly serrate margins. Both taxa exhibit overlapping flowering periods, though *C. konishii* initiates flowering approximately 1–2 weeks earlier in Taiwan's warmer subtropical climate. Mature trees of *C. konishii* reach heights of up to 50 m in high‐altitude forests (e.g., Dasyueshan, Taiwan), with a trunk diameter of 2.5 m at breast height. *C. lanceolata* are typically smaller, reaching 30 m in height under similar‐aged conditions, though trunk diameter (2.5–3 m) overlaps with *C. konishii*.

**TABLE 2:** Morphological differences between *C. konishii* and *C. lanceolata* .

Character	*Cunninghamia konishii*	*Cunninghamia lanceolata*
Bark color	Light reddish‐brown or reddish‐brown	Grayish‐brown with reddish lining
Needle morphology	leaves are lanceolate or strip‐lanceolate, usually slightly sickle‐shaped, radially extended	Leaves lanceolate or strip‐lanceolate, usually slightly curved and sickle‐shaped
Size	Up to 50 m, diameter at breast height 2.5 m (length 1.5–2 cm, width 1.5–2.5 mm)	Up to 30 m, diameter at breast height 2.5–3 m (length 2–6 cm, width 3–5 mm)
Cones	Oval or broadly ovate	Oval
Bracts	Bract scales ovate or long ovate, leathery, hard, apex triangular with a slightly acute, pointed tip, margin serrulate usually less conspicuous	Bract scales transversely elliptic, apex acute, distal margin membranous, irregularly denticulate, bract scales leathery when ripe, brownish‐yellow, triangular‐ovate, apex with hard, spiny cusp, margins irregularly serrate, revolute outward or not revolute

### 3.2 Genome‐Wide Discovery of SNPs

The sequencing of genomic complexity‐reduction libraries generated 916,398 tags with a minimum fragment length of 163 bp and a maximum of 314 bp, totaling 270,041,073 bp sequencing data. Additionally, 64.4% of the tags were 314 bp in length (Figure S3). Of the reads analyzed, a total of 833,789 SNP loci were included in all of the filtered samples. The average number of SNP loci per sample was 615,994, and the average proportion of SNP loci present in the population but missing in the individual was 26.12%. The filtering information for each sample, including the number of reads and the number of missing SNP loci, is provided in Table S1. The analysis of the number of private alleles in seven populations revealed the following results: the GD population had no private alleles, HN and SM each had one private allele, JX and NP each had eight private alleles, the TW population had 20 private alleles, and the HB population had the highest number of private alleles, totaling 59 (Table S2).

**FIGURE 2:**
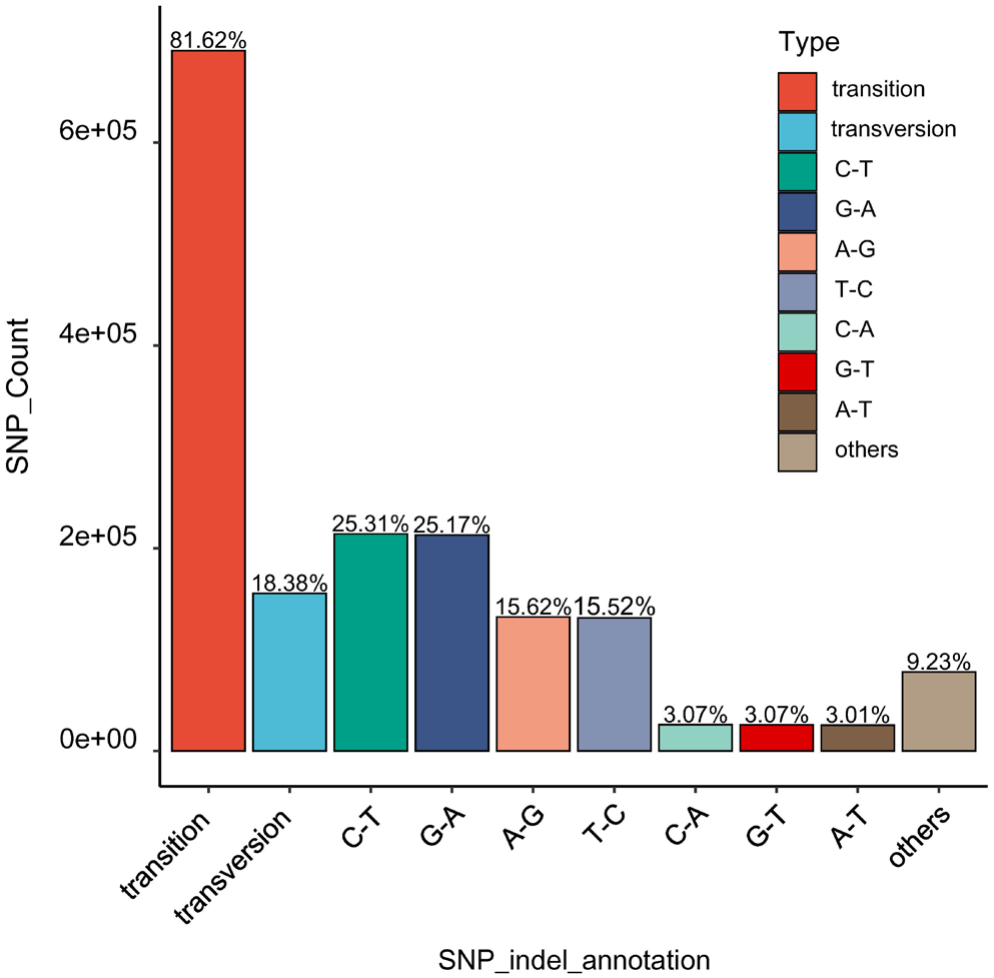
Transition and transversion of SNP sites.

Among the effective SNP sites obtained through simplified genome sequencing, 81.62% of the sites were single‐nucleotide transitions (Figure 2). Among them, the C/T transitions accounted for 25.31% of all SNP sites, the G/A transitions accounted for 25.17%, the A/G transitions accounted for 15.62%, and the T/C transitions accounted for 15.52%. Among all the SNP loci, 18.38% of the sites were single‐nucleotide transversions. Specifically, the C/A and G/T transversions accounted for 3.07% of all SNP loci, the A/T transversions accounted for 3.01%, and the remaining transversions accounted for 9.23%.

### 3.3 Genetic Diversity

Nucleotide diversity represents the genetic variation within a population. In this study, the SNP was used to calculate the average *θπ* between any two nucleotide sequences. Additionally, the genetic diversity of individuals within the population was analyzed by comparing the changes in *θπ* values. The analysis of intrapopulation genetic diversity revealed that the HB population, which is located in the marginal area of the northern boundary of Chinese fir, exhibited the smallest *θπ* value (4.828 × 10^−3^) and the lowest level of population genetic diversity. The TW population distributed in the high altitude area in the northern part of Taiwan Island had the largest *θπ* value (5.821 × 10^−3^) and the highest population genetic diversity. However, there was little overall difference in genetic diversity among the seven populations (Table 3). The Tajima's D values of each population were calculated by comparing the two mutation rates of *θπ* and *θw*. The Tajima's *D* values tended to be negative, indicating a positive selection effect. The results showed that the seven populations all exhibited varying degrees of environmental selection effects. The SM population had the highest Tajima's D value (−0.00835), but the smallest population GD value (−0.01847).

**TABLE 3:** Genetic diversity analysis of Chinese fir populations.

Population	SNP no.	*θπ*(10^−3^)	*θ*_*W*_ (10^−3^)	Tajima's *D*
GD	594,665	5.116	14.79	−0.01847
HB	601,785	4.828	12.807	−0.01118
HN	562,702	5.021	13.029	−0.0123
JX	661,187	5.635	12.737	−0.01
NP	609,162	5.588	12.67	−0.00969
SM	632,726	5.799	12.324	−0.00835
TW	670,412	5.821	14.425	−0.01613

Analysis of pairwise genetic differentiation and gene flow among the seven populations (Table 4) revealed that the highest value of genetic differentiation was observed between the HB and TW populations (*F*_ST_ = 0.122), while the smallest value was found between the GD and HN populations (*F*_ST_ = 0.045). The genetic differentiation between HB and the other six populations was greater than that among the six populations. However, the *F*_ST_ between TW and the six mainland China populations was slightly higher than that between the mainland Chinese fir populations. On the other hand, there was a significant level of gene flow between TW and mainland China populations, with an average *Nm* of 2.839. However, the gene flow between the TW and HB population was relatively weak, with an *Nm* of 1.799. The gene flow between GD and the other populations was relatively strong, while the gene flow between HB and the other populations was relatively weak. In general, *Nm* > 1 indicates that there is no significant genetic differentiation among populations. The low level of *F*_ST_ value, combined with a strong level of *Nm*, indicates a low differentiation among Chinese fir populations between mainland China and Taiwan Island.

**TABLE 4:** Genetic differentiation (*F*_ST_, upper right diagonal) and pairwise gene flow (*Nm*, down left diagonal) among the seven populations of Chinese fir.

Population	GD	HB	HN	JX	NP	SM	TW
GD	—	0.087	0.045	0.057	0.068	0.054	0.074
HB	2.625	—	0.092	0.109	0.116	0.103	0.122
HN	5.311	2.466	—	0.058	0.067	0.053	0.073
JX	4.149	2.047	4.036	—	0.074	0.061	0.079
NP	3.406	1.902	3.490	3.129	—	0.057	0.083
SM	4.359	2.173	4.470	3.824	4.150	—	0.072
TW	3.127	1.799	3.194	2.904	2.779	3.228	—

The directions of gene flow between mainland China groups and the TW population were analyzed using the Bayesian approach in Migrate to investigate the migration rates (Beerli and Palczewski 2010). The marginal likelihood of all three models supported a high probability of unidirectional migration from the mainland China population to the Taiwan population. This indicates that the level of gene flow from mainland China to Taiwan Island was more intense (Table 5). Therefore, this information indicates that the population of mainland China is more ancient than the population of Taiwan, and that a significant portion of the genetic makeup of TW population originated from the mainland China population by gene flow via the Dongshan Land Bridge.

**TABLE 5:** The marginal likelihoods of each probable evolutionary pattern between mainland China groups and the TW population.

Model	Raw thermodynamic score	Bezier approximated score	Harmonic mean
Mainland ⇆ TW	−383365.97	−330598.21	−322954.14
TW → Mainland	−384738.92	−329560.77	−321658.22
Mainland → TW	−370145.87	−318500.41	−312339.13

### 3.4 Population Structure

**FIGURE 3:**
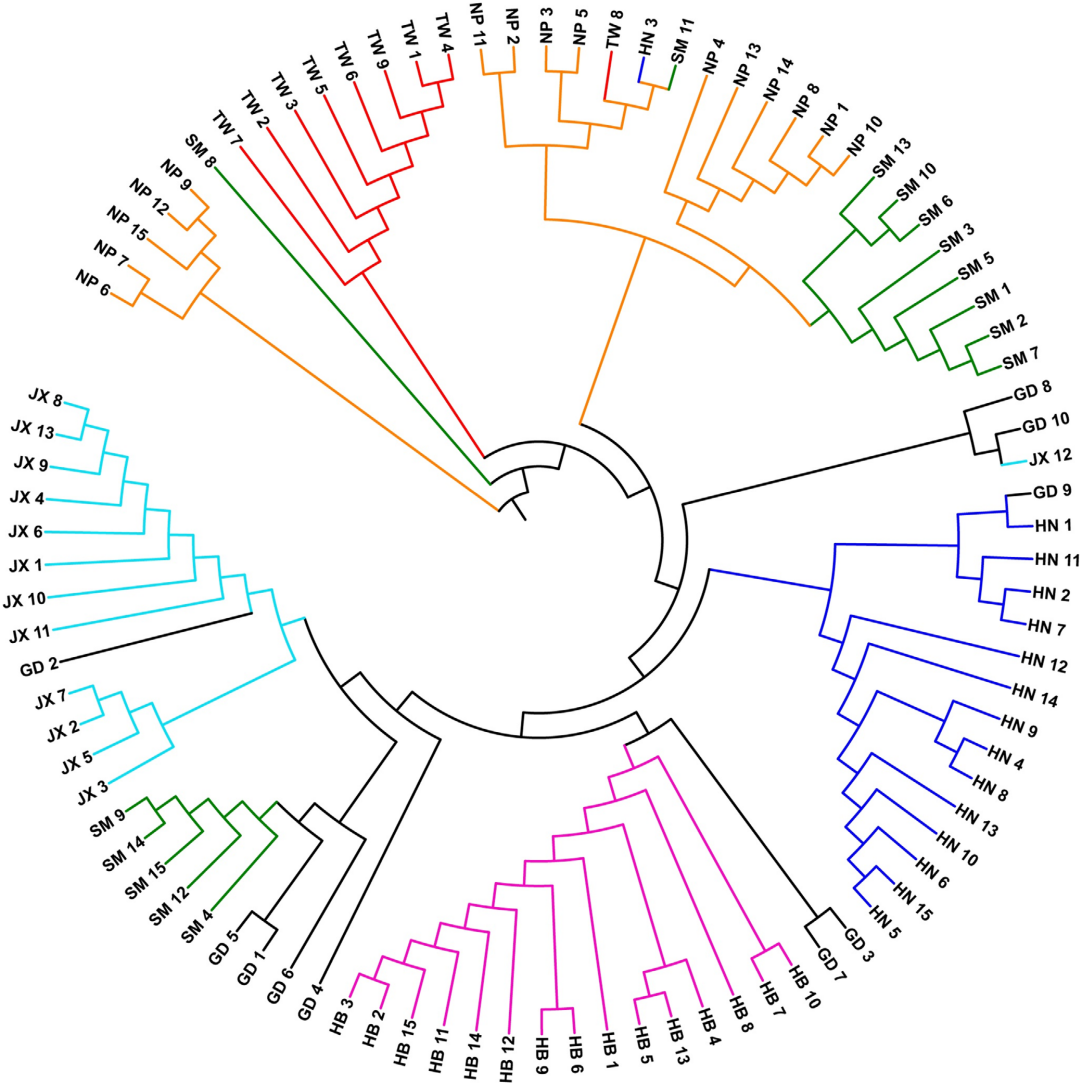
Neighbor‐joining clustering of the genetic distance tree of 92 accessions of Chinese fir.

A neighbor‐joining genetic distance tree of 92 accessions indicated that individuals from the same population were mostly clustered together, while accessions from the GD, SM, and NP populations were scattered across different branches of the tree (Figure 3). The accessions NP6, NP7, NP15, NP12, and NP9, which make up the NP population, clustered individually into one branch, while SM9 occupied a separate position in the tree. Ten accessions from NP and nine accessions from SM, both from Fujian province with a close geographical distance, were clustered into one branch. Meanwhile, 15 accessions from the HB population formed a distinct cluster that was separate from the others. Except for TW8, the accessions from TW clustered well into an independent clade, indicating their relatively consistent genetic background. Overall, most of the accessions from the same geographic origin were clustered together on the genetic distances tree and exhibited close kinship. The TW population and mainland China populations exhibited significant genetic differences and formed an independent branch on the genetic distances tree. The PCA clustering analysis also indicated that most samples clustered based on their geographic origin (Figure S4).

**FIGURE 4:**
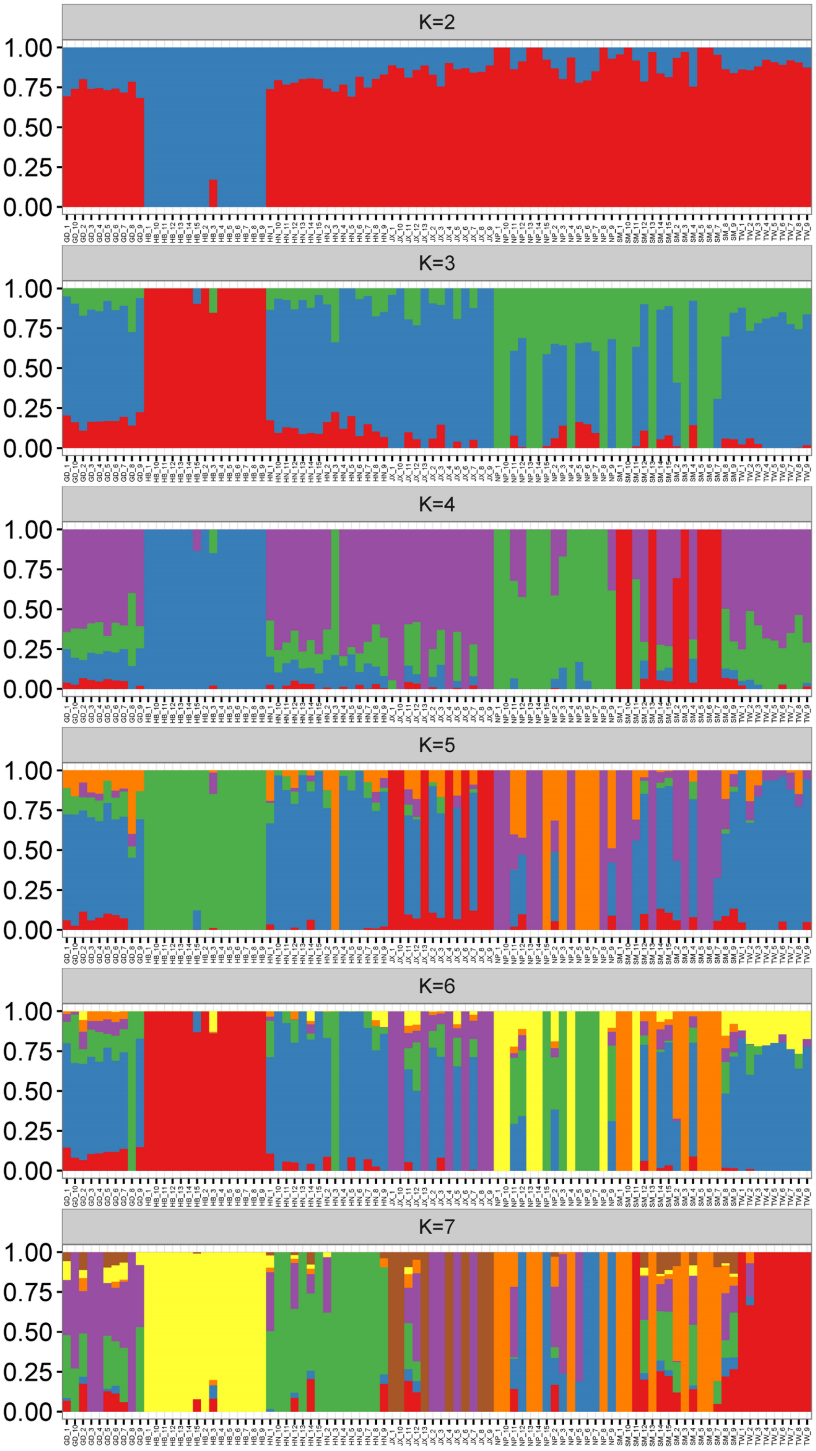
Population structure for 92 accessions is depicted in a clustering bar chart based on different population numbers (*K* = 2–7) generated by a Bayesian model algorithm and cross‐validation analysis.

To assess the structure of the Chinese fir diversity panel, the subgroups (optimal *K* value) for the population were determined using a Bayesian model algorithm and cross‐validation analysis. When the number of population subgroups was set to 1 (*K* = 1), the cross‐validation error rate was the lowest. This suggests that the optimal classification strategy would involve dividing the populations into 1 subgroup (Figure S5). This also indicates that the genetic structure of the seven Chinese fir populations from mainland China and Taiwan is closely related, with low differentiation. The population structure of the 92 accessions is most suitably divided into an overall group (Figure 4). The Bayesian‐based structure analysis at *K* = 2 clustered 15 accessions from the HB population together as one group and clustered the other accessions from mainland China and Taiwan together. At *K* = 3, six accessions from NP and eight accessions from SM clustered together. At *K* = 4, the lineage composition of GD, HN, JX, TW, and 4 accessions from SM cluster together with a probability of similar ancestry greater than 0.5. Additionally, the accessions of HN3 and SM1, along with 15 accessions from NP, cluster together. The eight accessions from SM and HB population cluster separately. As the value of *K* increased, the lineage composition of the 92 accessions became more mixed, with frequent intersections.

### 3.5 Correlation Analysis Between Genetic and Geographical Distances

**FIGURE 5:**
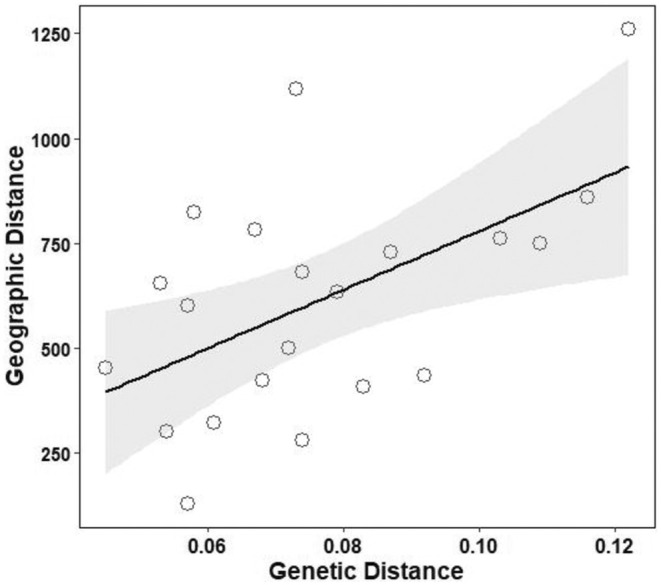
Relationship between genetic and geographic distances among Chinese fir populations.

**FIGURE 6:**
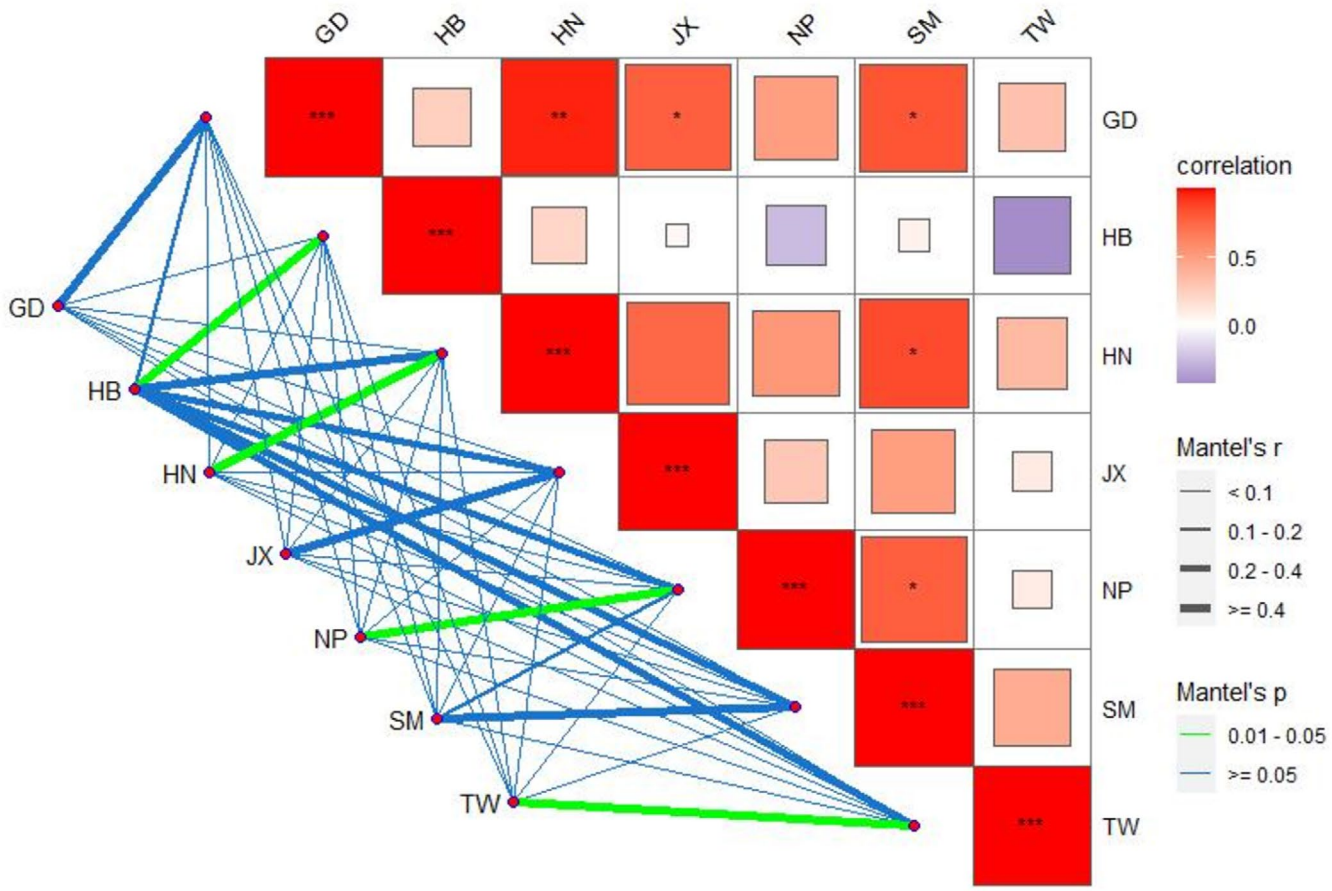
Mantel test of genetic distances and geographic distances among populations. The line thickness represents the correlation strength, and the color depth represents the *p* value.

The Mantel test was conducted on the matrices of geographic distance and genetic distance (Table S3). The results showed a significant positive correlation (*p* = 0.03; *r* = 0.545) between the geographic distance and genetic distance of different geographic groups of Chinese fir (Figure 5). Therefore, there is a relatively close genetic distance between Chinese fir populations that are geographically close to each other. Figure 6 shows the correlation between genetic and geographic distances among populations. The genetic distance of the HB, HN, NP, and TW populations was significantly correlated (*p* < 0.05) with geographic distance.

## 4 Discussion

### 4.1 Morphological Differences Between *C. lanceolata* and *C. konishii*

Previous studies revealed that *C. lanceolata* exhibits geographical variation in growth, sprouting, and wood properties, and that certain ecotypes such as *C. konishii*, Hubei weeping fir, and Dechang fir exist. The observed morphological variations of different ecotypes of *C. lanceolata* (e.g., leaf size, cone shape) fell within the range of intraspecific plasticity documented in widely distributed conifers (Chung et al. 2009; Shi et al. 2014). For instance, leaf dimensions in *C. lanceolata* correlate strongly with altitude and precipitation gradients across its range (Yan et al. 2021). Similarly, cone morphology differences may reflect adaptive responses to seed dispersal dynamics in Taiwan's high‐rainfall environments versus mainland China's drier habitats. The lack of clear morphological differentiation aligns with the low genetic divergence (*F*_ST_ = 0.045–0.122) and significant gene flow (*Nm* = 2.839) revealed by GBS analysis. These results suggest that historical isolation during glacial cycles, rather than reproductive barriers, drove the minor phenotypic divergence between populations. Consequently, the morphological distinctions do not warrant recognition of *C. konishii* as a separate species but support its classification as a regional variety of *C. lanceolata* . In the future, these ecotypes have the potential for further research to refine and enhance our understanding of the genetic differentiation of Chinese fir.

### 4.2 High‐Density SNP Discovery

SNPs have been proven to be the most abundant type of sequence variants in plant genomes, providing more detailed insights into genetic diversity and population structure (Yang et al. 2019). As a next‐generation sequencing (NGS)‐based SNP genotyping approach, the GBS is shown to be effective in discovering a large number of single‐nucleotide polymorphisms. This method is particularly suitable for population evolutionary analysis of conifers, which have large and complex genomes without reference genomes (Parchman et al. 2018; Zheng et al. 2019). Shirasawa et al. (2023) released the whole‐genome data of *C. lanceolata* obtained through PacBio HiFi sequencing. While whole‐genome sequencing provides comprehensive SNP coverage, GBS remains a cost‐effective and reliable method for population‐level studies, particularly in nonmodel species with large genomes. In this study, although GBS does not provide uniform genome‐wide coverage, the high density of SNPs (833,789 loci) ensures extensive representation of genetic variation across the genome. The number of SNP loci we discovered is abundant compared to the previous SNPs discovered in studies of Chinese fir that used the EcoR V‐based SLAF‐seq experiment (1,396,279 and 147,376 SNPs; Su et al. 2016; Zheng et al. 2019). Actually, SLAF‐seq is also a GBS method. Both methods provide a fast and cost‐effective way to generate genome‐wide SNPs, enabling us to decipher the genetic structure of conifer populations, such as Chinese fir (Ueno et al. 2019). We found that 81.62% of the obtained sites in the Chinese fir genome were single‐nucleotide site transitions, while only 18.38% were single‐nucleotide site transversions. This indicates a tendency for more transition substitutions than transversions in SNPs in the Chinese fir genome. The SLAF‐seq of Chinese fir also found a high estimated Ts:Tv ratio (3.15), indicating a deeper transition bias in Chinese fir (Zheng et al. 2019).

### 4.3 Genetic Diversity of *C. lanceolata* and *C. konishii*

The observed higher genetic diversity (θπ = 5.821 × 10^−3^) in the *C. konishii* (Taiwan) population compared to mainland *C. lanceolata* populations (e.g., θπ = 4.828 × 10^−3^ in Hubei) likely arises from a combination of historical, demographic, and environmental factors. Taiwan acted as a climatic refuge during Pleistocene glacial maxima, allowing *C. konishii* to retain ancestral genetic diversity. In contrast, mainland populations experienced repeated bottlenecks during glacial expansions, reducing diversity. This aligns with phylogeographic patterns in other East Asian gymnosperms (Chou et al. 2011; Shi et al. 2014). While gene flow is unidirectional (mainland → Taiwan), the Taiwan population likely received migrants from diverse mainland lineages via the Dongshan Land Bridge. This “melting pot” effect blended genetic variation from multiple sources, elevating *θπ*. In contrast, mainland populations are more isolated from one another (higher *F*_*ST*_ among them), leading to lower within‐population diversity. Taiwan's steep elevational gradients and diverse microclimates support heterogeneous selection pressures, maintaining a broader array of alleles. Mainland populations, occupying more uniform subtropical habitats, may experience stronger selective sweeps, reducing diversity. The Taiwan population, located in the protected Dasyueshan National Forest, has likely retained a larger diversity and older alleles. *Tajima's D* values (negative across all populations) suggest recent population expansions. However, Taiwan's less severe post‐glacial expansion (due to its refugial status) may have preserved more ancient polymorphisms, whereas mainland populations underwent stronger founder effects during recolonization. Taiwan's higher *θπ* reflects a greater proportion of “shared polymorphisms” maintained across individuals, indicative of long‐term stability and gene flow.

### 4.4 Genetic Differentiation Mechanisms of *C lanceolata* and *C. konishii*

The near‐zero values of *Tajima's D* observed in our study (ranging from −0.00835 to −0.01847) do indeed align with expectations under neutral evolution, where genetic drift dominates over selection. The slight negative skew in Tajima's D aligns with “post‐glacial population expansion,” a common pattern in East Asian conifers (Shi et al. 2014). The absent or week selection signal maybe contribute to the long generation times of Chinese fir, the slow substitution rates in Cunninghamia reduce the footprint of selection over short timescales. Although the Tajima's D value is close to 0, suggesting that the selection effect is not significant, the document notes that all populations exhibit varying degrees of environmental selection effects. Moreover, the higher *θw* value (14.425 × 10^−3^) for the TW population further supports the hypothesis that it has experienced a certain degree of positive selection. While our sample sizes are sufficient for population‐level inferences, larger samples (e.g., > 30 individuals/population) would improve resolution of subtle selection signals. Whole‐genome sequencing could further distinguish neutral and adaptive variation.

The observed *F*_*ST*_ value of 0.122 between the HB and TW populations reflects moderate genetic differentiation, consistent with partial isolation and limited gene flow. During interglacial periods (e.g., the Holocene), rising sea levels submerged the Dongshan Land Bridge, physically isolating the TW population from mainland populations like HB. This geographic barrier reduced contemporary gene flow, promoting divergence. During glacial maxima, mainland populations migrated southward, including to Taiwan via the Dongshan Land Bridge. However, post‐glacial recolonization of northern regions (e.g., HB) likely involved founder effects, reducing genetic diversity and increasing divergence from TW. The HB population resides in the mountainous regions of Hubei Province (1207 m elevation), while TW occupies high‐altitude forests (2250 m). HB experiences a cooler, drier climate (10.2°C, 1550 mm precipitation) compared to TW (12.4°C, 3800 mm). These divergent selective pressures may favor locally adapted alleles, though *Tajima's D* values suggest that neutrality dominates. TW's high‐altitude environment may select for traits distinct from HB populations. Migration analysis revealed asymmetric gene flow from mainland to Taiwan, but minimal reverse migration. This unidirectional flow introduced mainland alleles into TW but did not homogenize populations due to geographic isolation. The gene flow estimate between HB and TW (*Nm* = 1.799) is lower than the average *Nm* across other mainland‐TW pairs (2.839). Thus, we believe that HB, as a marginal population, lies at the northern edge of the range of *C. lanceolata* , where smaller effective population sizes and historical bottlenecks (evidenced by low *θπ* = 4.828 × 10^−3^) amplify drift‐driven differentiation. Taiwan's stable climate during glacials preserved higher diversity (*θπ* = 5.821 × 10^−3^), but limited post‐glacial gene flow maintained distinctiveness from mainland populations. This pattern aligns with phylogeographic studies of East Asian gymnosperms (Ho et al. 2014; Chou et al. 2011), where land bridges and glacial cycles shape differentiation.

The near‐negligible differentiation between GD and HN arises from their geographic proximity, high gene flow, shared post‐glacial history, and environmental homogeneity. This contrasts with moderately divergent populations (e.g., HB‐TW), where barriers like the Taiwan Strait amplify differentiation. GD and HN are adjacent provinces in southern China, separated by short distances (Table 1: GD at 24.75°N, HN at 26.25°N). Their proximity facilitates “ongoing gene flow” via pollen and seed dispersal, which homogenizes genetic variation. The pairwise *Nm* between GD and HN is 5.311, the highest among all population pairs. This indicates frequent historical and contemporary gene exchange, effectively counteracting genetic drift. Thus, we hypothesis GD and HN likely originated from the same glacial refugia in southern China (e.g., Jiangxi/Guizhou; An 2012). Post‐glacial northward expansion into Guangdong and Hunan would have involved shared ancestral lineages, minimizing divergence. Negative *Tajima's D* values (GD: −0.01847; HN: −0.0123) suggest that both populations underwent “recent expansions,” maintaining similar allele frequency distributions. The *F*_*ST*_ of GD‐HN (0.045) is significantly lower than the HB‐TW (0.122), underscoring the role of “geographic isolation” in driving divergence between distant populations (e.g., Hubei and Taiwan) versus connectivity in proximate ones (GD‐HN).

### 4.5 Taxonomic Analysis of *C. lanceolata* and *C. konishii*

The study on the genetic relationships and phytogeography of the Chinese fir populations in China and Taiwan is interesting, and the taxonomy remains controversial. Lin (2000) used cpDNA markers to analyze it and concluded that *C. konishii* from Taiwan had evolved from *C. lanceolata* in mainland China and gradually developed into a new species. Hwang et al. (2003) believed that the population of *C. konishii* originated from mainland China through long‐distance seed dispersal after the ice age. Other studies have also concluded that there is a very close genetic relationship between C. konishii and *C. lanceolata* , suggesting that they are the same species (Chung et al. 2004; Chung et al. 2009). Using cpDNA markers, a genealogical geographic analysis was conducted on the germplasm of the Chinese fir provenance test forests established in China in 1981. The analysis revealed that during the ice age, the refuge of Chinese fir was located in Jiangxi, Guizhou, and Yunnan (An 2012). Moreover, its population genetic differentiation was much higher than that reported by other researchers (Hwang et al. 2003). Using ISSR markers, lower levels of genetic diversity were observed in both Fujian province (*h* = 0.325) and the Taiwan area (*h* = 0.263) (Chen et al. 2017). Our results also confirmed that there is little difference in the genetic diversity of Chinese fir between mainland China and Taiwan. Additionally, the genetic diversity of Chinese fir was found to be low. Other studies have also demonstrated a low level of genetic diversity in Chinese fir populations using various molecular markers such as SRAP, ISSR, and SSR, among others. It is worth noting that mainland China only has one species of *Cunninghami*a (Duan et al. 2015; Huang et al. 2022; Li et al. 2017).

Geographic barriers between islands and nearby continents provide an opportunity to study patterns of differentiation between island populations and neighboring continental populations through intermittent gene flow (Zheng et al. 2019; Papachristou et al. 2020). Dongshan Land Bridge (also known as the Min‐Taiwan Land Bridge) connected the two sides of the strait during the glacial period. The water depth did not exceed 40 m during the interglacial period (Cui et al. 2011; Zhao et al. 2017). The isolation of gymnosperms in mainland China and Taiwan led to their isolation and differentiation, resulting in the emergence of new species or varieties (Huang 2014; Lin and Chung 2017). Due to differences in evolutionary rates and levels of gene exchange, certain intraspecific or interspecific heterologous plant populations have differentiated into new species, varieties, or subspecific variants in Taiwan and southwest China, respectively (Shi et al. 2014). For example, the genus *Keteleeria* has differentiated into *Keteleeria fortunei* in mainland China and *Keteleeria davidiana* in Taiwan. Similarly, the genus *Amentotaxus* has differentiated into *Amentotaxus yunnanensis* and *Amentotaxus formosana* (Ho et al. 2014). However, *Taiwania cryptomerioides* remained the same species in both sites of the Strait (Chou et al. 2011; Ho et al. 2014).

Therefore, the *F*_*ST*_ between Taiwan (*C. konishii*) and mainland populations (range: 0.072–0.122) falls within “intraspecific” divergence levels for conifers (e.g., *Pinus sylvestris* , *F*_*ST*_ = 0.05–0.15; Hall et al. 2020). High level of gene flow (*Nm* = 2.839) and unidirectional migration from mainland to Taiwan indicates historical connectivity and ongoing reproductive compatibility. Population Structure and PCA (Figures 4, 5 and S4) show overlapping genetic clusters, with no distinct lineage separating “*C. konishii*” from mainland populations. Current evidence does not justify retaining *C. konishii* as a distinct species. Although there are some morphological differences, traits like leaf size and cone morphology are variable within widespread conifer species. Varietal status is common for geographically isolated populations with minor morphological distinctions (e.g., *Juniperus osteosperma* var. *utahensis*). Thus, *C. konishii* is not an independent species, we propose reclassifying “*C. konishii*” as “ *C. lanceolata* var. *Konishii*.” The reason for this may be that the Dongshan Land Bridge, which connected the two sides of the Taiwan Strait, existed for a significant period of time before being submerged approximately 20,000 years ago (Zhao et al. 2017; Zhao et al. 2018). The gene exchange of Chinese fir on both sides of the Taiwan Strait was extensive, and the period of geographic isolation was relatively brief. However, the environmental selection pressures on Chinese fir on both sides of the Taiwan Strait were not significant enough to accumulate sufficient genetic variation for species differentiation. In addition, the natural distribution area of Chinese fir in Taiwan is close to the area where it is artificially cultivated. Furthermore, most of the germplasm resources of Chinese fir in Taiwan come from mainland China, specifically from Fujian and Guangdong. The gene exchange between the natural population and artificial population of Chinese fir in Taiwan may also have a certain impact on the population genetic structure and species differentiation of Chinese fir in mainland China and Taiwan. Moreover, experimental introduction of *C. konishii* to a mainland germplasm bank demonstrated its capacity to produce viable offspring under natural pollination conditions adjacent to *C. lanceolata* . As a whole, the results of this study elucidate the historical dynamics of Chinese fir populations and the mechanisms of species evolution. They also reveal the important role of the Dongshan Land Bridge in gene exchange and species differentiation. Additionally, the study analyzes the floristic distribution pattern and the formation mechanism of plant diversity in Taiwan.

## 5 Conclusions

This study on the species differentiation of Chinese fir in mainland China and Taiwan provides valuable insights into gene exchange and species evolution. Using the GBS method, we identified 833,789 SNPs, 82% of which were single‐nucleotide transitions, indicating a notable transition bias in Chinese fir. The northern marginal population (HB) of *C. lanceolata* exhibited the lowest genetic diversity, while the Taiwan population of *C. konishii*, found in northern high‐altitude areas, exhibited the highest genetic diversity. Overall, genetic diversity was generally low, with minimal differences between populations in mainland China and Taiwan. The *F*_*ST*_ values (0.072–0.122) fall within the expected “intraspecific” divergence range for conifers, and a strong level of gene flow (*Nm* = 2.839) suggests a low differentiation among Chinese fir populations in mainland China and Taiwan, which is supported by a high probability of unidirectional migration from mainland China to Taiwan, with the Dongshan Land Bridge facilitating pre‐glacial gene flow. PCA and population structure analyses show overlapping genetic clusters, with no clear separation between C. konishii and mainland populations. Combined with genomic evidence of historical gene flow and low genetic differentiation, we conclude that *C. konishii* represents an ecotype of *C. lanceolata* , shaped by environmental plasticity and incomplete isolation.

## Author Contributions

**Yajing Zhang:** conceptualization (equal), data curation (lead), writing – original draft (equal). **Yangyang Sun:** conceptualization (equal), investigation (lead), writing – original draft (equal). **Minchen Zhong:** methodology (lead). **Fenglin Chen:** formal analysis (equal). **Yaning Wang:** formal analysis (equal). **Mulualem Tigabu:** writing – review and editing (equal). **XiangQing Ma:** validation (lead), writing – review and editing (equal). **Ming Li:** project administration (lead), supervision (lead), writing – review and editing (equal).

## Conflicts of Interest

The authors declare no conflicts of interest.

## Supporting information


**Figure S1.** The sampled trees of *Cunninghamia konishii* from Dasyueshan, Taiwan (left) and *Cunninghamia lanceolata* from Nanping, Fujian Province (right).
**Figure S2**. Hand‐drawn pictures of *Cunninghamia konishii (A) and Cunninghamia lanceolata* (B).
**Figure S3**. Length distribution of assembly contigs.
**Figure S4**. PCA analysis of 92 accessions.
**Figure S5**. Cross‐Validation error analysis of population structure.
**Table S1**. Reads filter information and SNP quantity information of every sample.
**Table S3**. Geographic distance among the seven populations of Chinese fir.

**Table S2.** Number of private alleles in populations.

## Data Availability

The original sequencing data of 92 accessions by GBS sequencing were deposited in the NCBI SRA database under the accession number of PRJNA951606. The data was released and can be accessible with the following link: https://www.ncbi.nlm.nih.gov/sra/PRJNA951606.
