# Supplementary material for: Genetic Differentiation of Chinese Fir Populations From Mainland China and Taiwan as Revealed by Genotyping‐By‐Sequencing Analysis, With Implication for Taxonomic Position of the Species
Source: Ecol Evol. 2025 Apr 11;15(4):e71270. doi: 10.1002/ece3.71270 (PMC11992008; doi:10.1002/ece3.71270)
Supplement: Supplementary file 1 — Figure S1. The sampled trees of Cunninghamia konishii from Dasyueshan, Taiwan (left) and Cunninghamia lanceolata from Nanping, Fujian Province (right). Figure S2. Hand‐drawn pictures of Cunninghamia konishii (A) and Cunninghamia lanceolata (B). Figure S3. Length distribution of assembly contigs. Figure S4. PCA analysis of 92 accessions. Figure S5. Cross‐Validation error analysis of population structure. Table S1. Reads filter information and SNP quantity information of every sample. Table S3. Geographic distance among the seven populations of Chinese fir. [file ECE3-15-e71270-s001.docx]

**Table S1.** Reads filter information and SNP quantity information of every sample.

| Sample | Clean Reads numbers | High Quantity Clean Reads Numbers | SNP Numbers | Missing SNP Numbers | Missing Ratio |
| --- | --- | --- | --- | --- | --- |
| GD_1 | 15921640 | 15854076 | 615138 | 218651 | 26.22% |
| GD_2 | 14663870 | 14605894 | 650924 | 182865 | 21.93% |
| GD_3 | 14453662 | 14380758 | 602507 | 231282 | 27.74% |
| GD_4 | 15509782 | 15446304 | 606804 | 226985 | 27.22% |
| GD_5 | 15436644 | 15359322 | 642854 | 190935 | 22.90% |
| GD_6 | 16240538 | 16164708 | 658220 | 175569 | 21.06% |
| GD_7 | 12204724 | 12139908 | 575827 | 257962 | 30.94% |
| GD_8 | 10238214 | 10189438 | 488957 | 344832 | 41.36% |
| GD_9 | 10854294 | 10813158 | 492413 | 341376 | 40.94% |
| GD_10 | 14381444 | 14300732 | 613004 | 220785 | 26.48% |
| HB_1 | 11430878 | 11355160 | 562725 | 271064 | 32.51% |
| HB_2 | 22322114 | 22169878 | 726090 | 107699 | 12.92% |
| HB_3 | 23329282 | 23077700 | 739494 | 94295 | 11.31% |
| HB_4 | 9341868 | 9280060 | 482477 | 351312 | 42.13% |
| HB_5 | 10508974 | 10454266 | 488806 | 344983 | 41.38% |
| HB_6 | 12785238 | 12685344 | 588338 | 245451 | 29.44% |
| HB_7 | 11716926 | 11617960 | 580013 | 253776 | 30.44% |
| HB_8 | 12416438 | 12303446 | 558712 | 275077 | 32.99% |
| HB_9 | 12836294 | 12746176 | 595061 | 238728 | 28.63% |
| HB_10 | 11626554 | 11527028 | 576771 | 257018 | 30.83% |
| HB_11 | 16257072 | 16143668 | 656591 | 177198 | 21.25% |
| HB_12 | 15301248 | 15200790 | 640469 | 193320 | 23.19% |
| HB_13 | 11884646 | 11799000 | 489017 | 344772 | 41.35% |
| HB_14 | 16059106 | 15954550 | 637946 | 195843 | 23.49% |
| HB_15 | 17940326 | 17837910 | 704265 | 129524 | 15.53% |
| HN_1 | 11825700 | 11756044 | 545628 | 288161 | 34.56% |
| HN_2 | 13326718 | 13250296 | 579815 | 253974 | 30.46% |
| HN_3 | 14106078 | 13981200 | 226082 | 607707 | 72.88% |
| HN_4 | 11154614 | 11097368 | 559230 | 274559 | 32.93% |
| HN_5 | 13226606 | 13145232 | 563134 | 270655 | 32.46% |
| HN_6 | 13207680 | 13107124 | 630314 | 203475 | 24.40% |
| HN_7 | 11569032 | 11476692 | 561183 | 272606 | 32.69% |
| HN_8 | 10850506 | 10772234 | 569719 | 264070 | 31.67% |
| HN_9 | 12003770 | 11922656 | 621804 | 211985 | 25.42% |
| HN_10 | 12079750 | 11983196 | 604847 | 228942 | 27.46% |
| HN_11 | 9957646 | 9886442 | 549484 | 284305 | 34.10% |
| HN_12 | 10848430 | 10772358 | 584977 | 248812 | 29.84% |
| HN_13 | 9882532 | 9828644 | 591490 | 242299 | 29.06% |
| HN_14 | 11439298 | 11359680 | 634510 | 199279 | 23.90% |
| HN_15 | 12354568 | 12276832 | 618310 | 215479 | 25.84% |
| JX_1 | 11345512 | 11284702 | 609959 | 223830 | 26.84% |
| JX_2 | 17665812 | 17573146 | 687710 | 146079 | 17.52% |
| JX_3 | 13666258 | 13588178 | 596580 | 237209 | 28.45% |
| JX_4 | 16262112 | 16182422 | 684546 | 149243 | 17.90% |
| JX_5 | 13939058 | 13858018 | 637499 | 196290 | 23.54% |
| JX_6 | 16417160 | 16324192 | 670346 | 163443 | 19.60% |
| JX_7 | 18418452 | 18311246 | 716488 | 117301 | 14.07% |
| JX_8 | 18040508 | 17937882 | 701523 | 132266 | 15.86% |
| JX_9 | 15127094 | 15034884 | 680650 | 153139 | 18.37% |
| JX_10 | 11242142 | 11175894 | 567600 | 266189 | 31.93% |
| JX_11 | 14375420 | 14285210 | 639154 | 194635 | 23.34% |
| JX_12 | 16021126 | 15920002 | 680408 | 153381 | 18.40% |
| JX_13 | 19588876 | 19464836 | 722971 | 110818 | 13.29% |
| NP_1 | 17976120 | 17818652 | 694074 | 139715 | 16.76% |
| NP_10 | 16498646 | 16371476 | 654602 | 179187 | 21.49% |
| NP_11 | 11513936 | 11419406 | 557360 | 276429 | 33.15% |
| NP_12 | 28421972 | 28196064 | 782956 | 50833 | 6.10% |
| NP_13 | 12192284 | 12058462 | 610042 | 223747 | 26.83% |
| NP_14 | 13483212 | 13356702 | 631595 | 202194 | 24.25% |
| NP_15 | 11689974 | 11583764 | 587578 | 246211 | 29.53% |
| NP_2 | 11428232 | 11350764 | 590458 | 243331 | 29.18% |
| NP_3 | 13334112 | 13194100 | 547998 | 285791 | 34.28% |
| NP_4 | 9974500 | 9860100 | 448217 | 385572 | 46.24% |
| NP_5 | 11924152 | 11794736 | 485633 | 348156 | 41.76% |
| NP_6 | 18742386 | 18522274 | 588408 | 245381 | 29.43% |
| NP_7 | 14153908 | 14025738 | 552239 | 281550 | 33.77% |
| NP_8 | 16178948 | 16045362 | 649028 | 184761 | 22.16% |
| NP_9 | 25898320 | 25699968 | 757249 | 76540 | 9.18% |
| SM_1 | 18777714 | 18603588 | 684562 | 149227 | 17.90% |
| SM_2 | 24002048 | 23745440 | 762594 | 71195 | 8.54% |
| SM_3 | 13568002 | 13465070 | 618427 | 215362 | 25.83% |
| SM_4 | 14690084 | 14547682 | 647428 | 186361 | 22.35% |
| SM_5 | 15133646 | 15041792 | 669958 | 163831 | 19.65% |
| SM_6 | 10465806 | 10382628 | 551653 | 282136 | 33.84% |
| SM_7 | 18716874 | 18588898 | 721901 | 111888 | 13.42% |
| SM_8 | 16303788 | 16191316 | 658180 | 175609 | 21.06% |
| SM_9 | 14326524 | 14214768 | 693424 | 140365 | 16.83% |
| SM_10 | 10182136 | 10118806 | 533721 | 300068 | 35.99% |
| SM_11 | 6374876 | 6324502 | 399087 | 434702 | 52.14% |
| SM_12 | 16037042 | 15896760 | 682046 | 151743 | 18.20% |
| SM_13 | 10695088 | 10611840 | 512204 | 321585 | 38.57% |
| SM_14 | 14296934 | 14189708 | 693023 | 140766 | 16.88% |
| SM_15 | 13378132 | 13276862 | 662680 | 171109 | 20.52% |
| TW_1 | 25053156 | 24875804 | 761710 | 72079 | 8.64% |
| TW_2 | 15311824 | 15193720 | 634333 | 199456 | 23.92% |
| TW_3 | 14984780 | 14883908 | 648297 | 185492 | 22.25% |
| TW_4 | 16945308 | 16836818 | 703659 | 130130 | 15.61% |
| TW_5 | 15540402 | 15428646 | 694690 | 139099 | 16.68% |
| TW_6 | 18313978 | 18184054 | 733449 | 100340 | 12.03% |
| TW_7 | 12356278 | 12280124 | 603765 | 230024 | 27.59% |
| TW_8 | 9892634 | 9852262 | 529042 | 304747 | 36.55% |
| TW_9 | 17103638 | 16967924 | 724764 | 109025 | 13.08% |

**Table S3.** Geographic distance among the seven populations of Chinese fir.

| Population | HB | HN | JX | NP | SM | TW |
| --- | --- | --- | --- | --- | --- | --- |
| GD | 727.503 | 451.394 | 600.051 | 421.615 | 302.236 | 680.496 |
| HB |  | 436.010 | 749.666 | 859.211 | 762.764 | 1260.751 |
| HN |  |  | 824.171 | 782.224 | 654.548 | 1117.706 |
| JX |  |  |  | 280.764 | 323.048 | 634.708 |
| NP |  |  |  |  | 129.339 | 407.444 |
| SM |  |  |  |  |  | 499.523 |

**
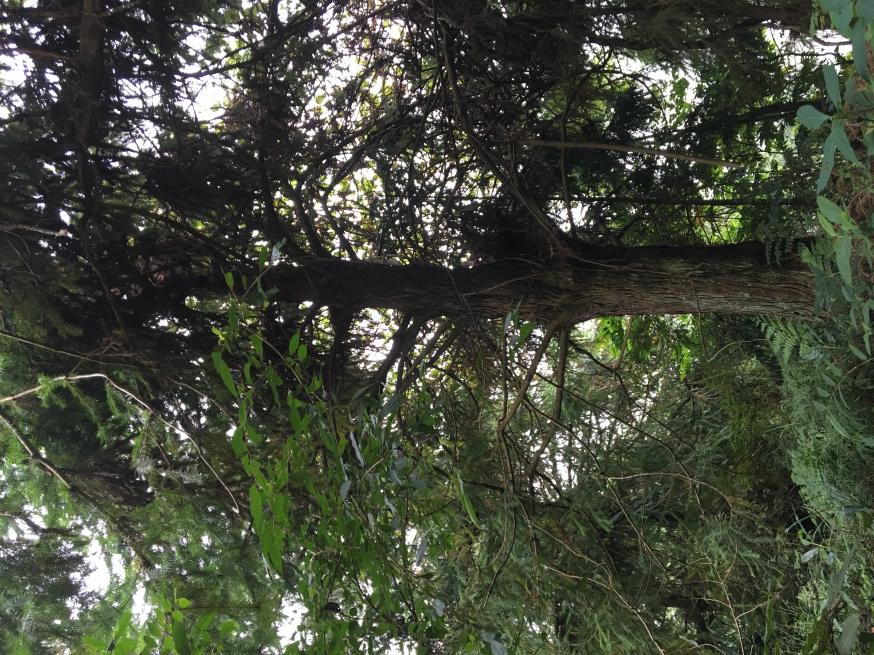

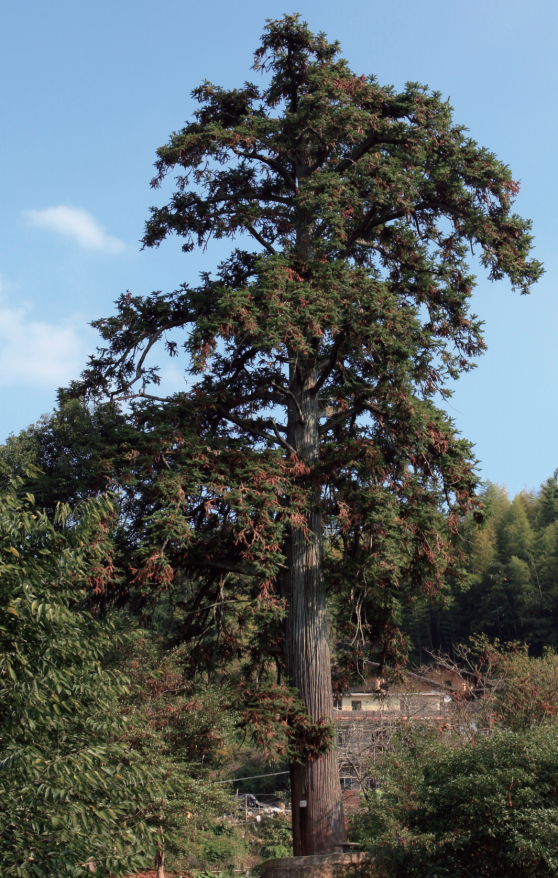
**

**Figure S1.** The sampled trees of *Cunninghamia* *konishii* from Dasyueshan, Taiwan (left) and *Cunninghamia lanceolata* from Nanping, Fujian Province (right)


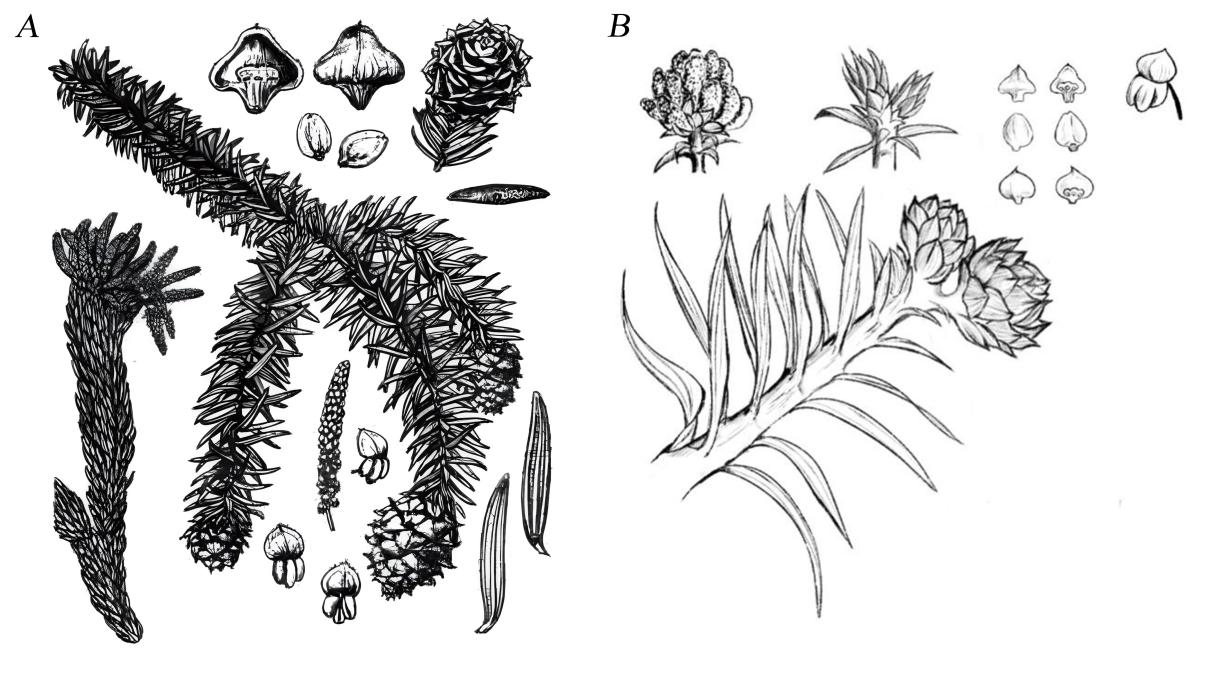


**Figure S2.** Hand-drawn illustration of *Cunninghamia konishii* (A) and *Cunninghamia lanceolata* sample trees from Nanping, Fujian Province (B).

(Figure S2A is modified from Li Hui-Lin. 1975. Flora of Taiwan, V.1, parts 1-8. Taipei: Epoch Publishing.

For an intuitive understanding of the morphology of *Cunninghamia konishii*, please click the link: https://www.tbn.org.tw/sites/ozop/files/styles/extra_large/public/occurrence/media/62/f5/file-9190b3af-0af4-422c-93ee-df57e5242d70_0.jpeg?itok=fp_5hhb0. )


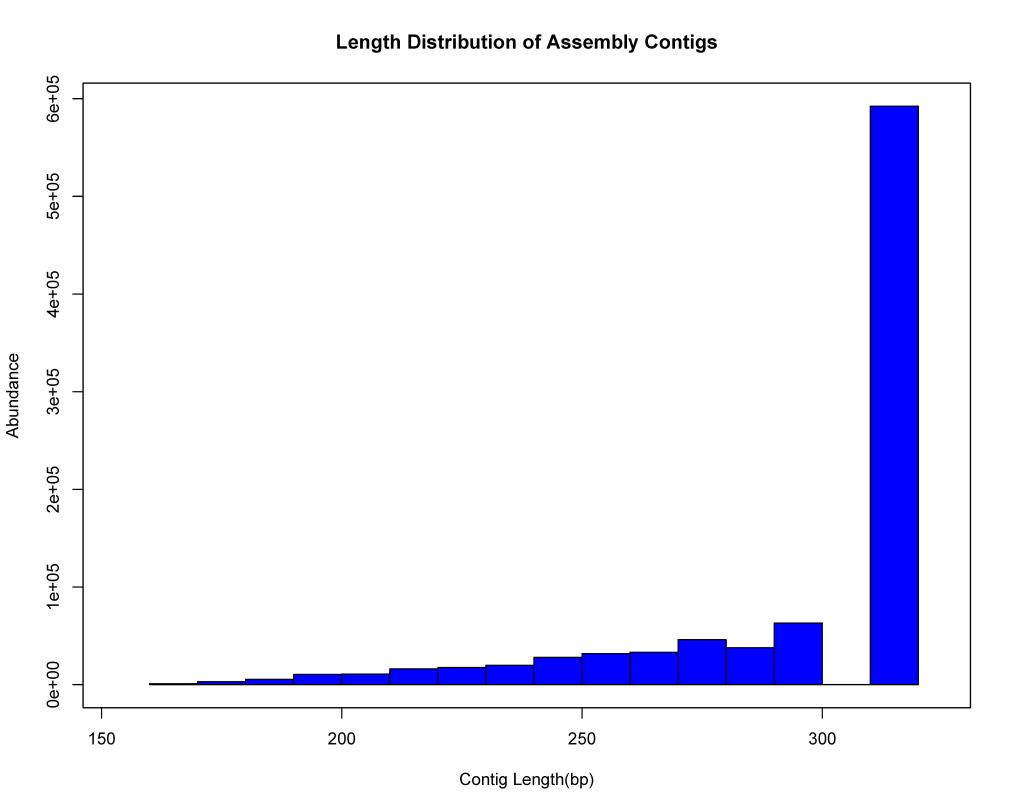


**Figure S3.** Length distribution of assembly contigs.


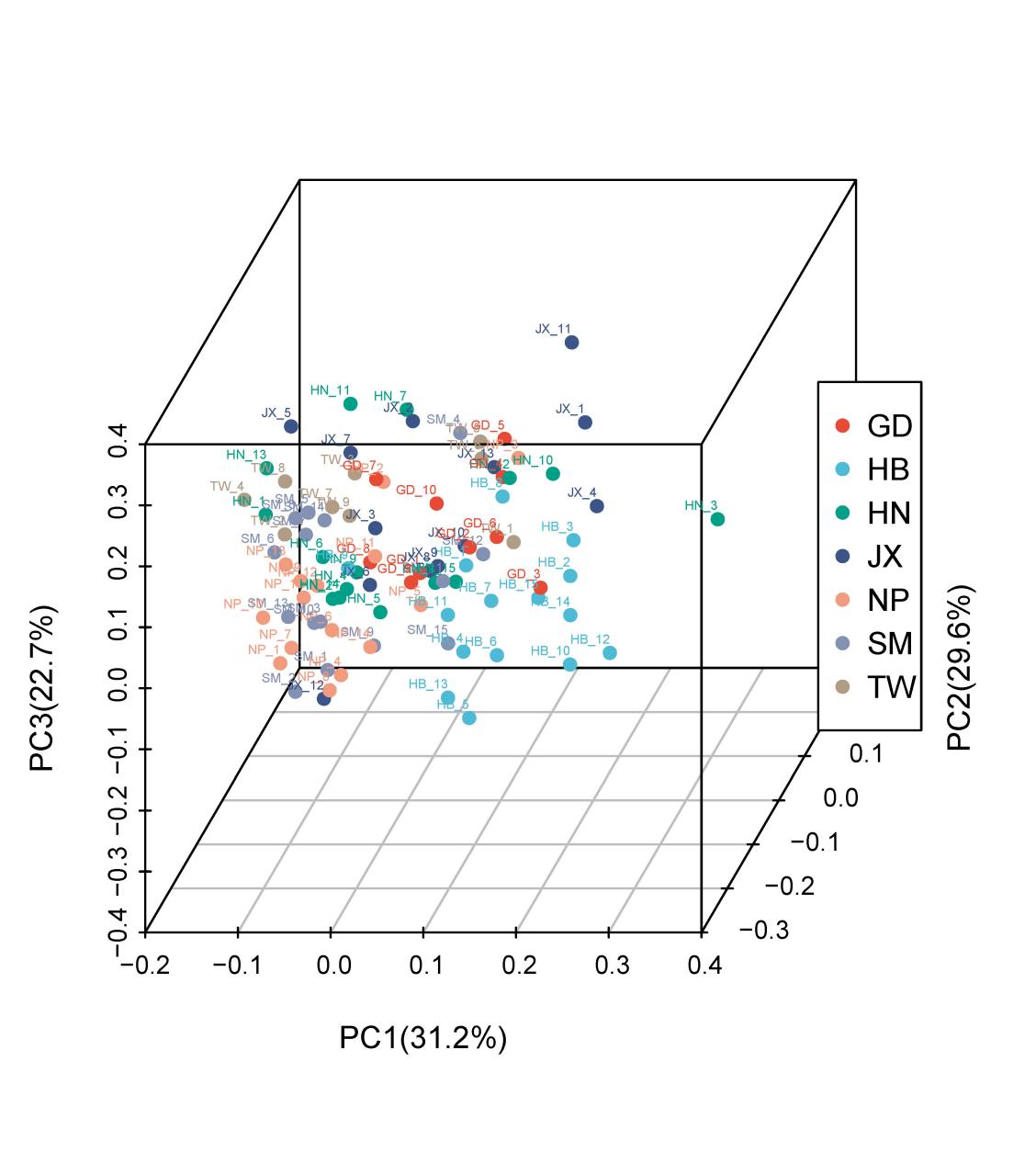


**Figure S4.** PCA analysis of 92 accessions.


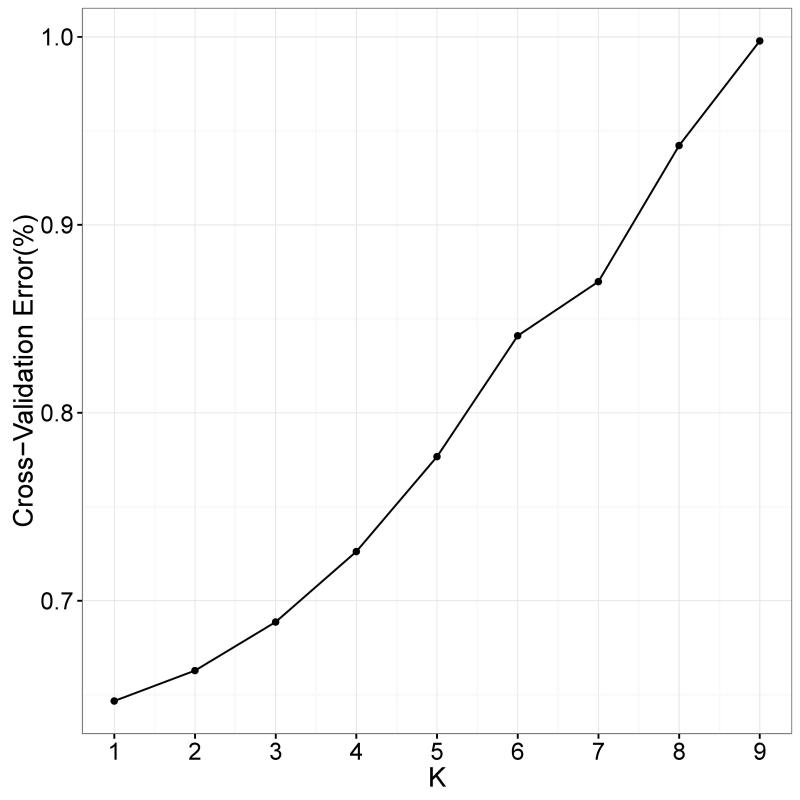


**Figure S5.** Cross-Validation error analysis of population structure.
